# Supplementary material for: Strategies to address recruitment to a randomised trial of surgical and non-surgical treatment for cancer: results from a complex recruitment intervention within the Mesothelioma and Radical Surgery 2 (MARS 2) study
Source: BMJ Open. 2024 May 16;14(5):e079108. doi: 10.1136/bmjopen-2023-079108 (PMC11103236; doi:10.1136/bmjopen-2023-079108)
Supplement: Supplementary data [file bmjopen-2023-079108supp001.pdf]

Supplement 1: QRI-Informed Recruitment Tips document

Nicola.mills@bristol.ac.uk

Recruitment and Informed Consent Guidance

March 2019

This document includes suggestions that can help with recruitment and informed consent. You may wish to consider using them alongside your own individual style.

Introducing MARS 2

- Approach all potentially eligible patients so that everyone has an opportunity to consider participation (including those with biphasic and sarcomatoid disease).
- Mention the study early on and explain the equipoise *('Because we don't know if chemotherapy on its own or chemotherapy with surgery is best we're taking part in a study called MARS 2')*. Be mindful to convey equipoise throughout.
- Request patients to *'keep an open mind'* until all information is heard.

Discussing the study

- Present MARS 2 in an enthusiastic and straight-forward manner.
- It is better to use the term 'study' rather than 'trial' as trial means different things to different patients and can therefore be confusing *(e.g. 'trial and error', 'experimental', 'guinea pig')*.
- It can be good to mention that MARS 2 is a study funded by the NIHR – the NHS funding body - and is being carried out in over 25 centres around the UK.
- Describe the benefits of study participation, e.g. close follow-up and monitoring, and that the aim of research is to produce evidence so that future patients will not have to face current treatment uncertainties.
- Be clear that participation is voluntary and that their care will not be affected in anyway if they choose not to participate.

Balancing the treatments

- You are not expected to give detailed information about chemotherapy or surgery if you do not specialise in it, but you can still ensure that you convey equipoise throughout the consultation when explaining treatments:
  - Remind patients that we do not know if chemotherapy on its own or in combination with surgery is best, hence the need for the study.
  - When outlining the treatments think 'balance' – are you inadvertently steering patients?
    - Avoid loaded terminology (i.e. 'gold standard', 'experimental')
    - Balance the potential advantages and disadvantages of the treatments e.g.

      "Those are the risks of surgery, but then as I said it comes back to that balance of risks and benefits – what are the potential benefits of surgery with chemotherapy…. How then does this compare with the chemotherapy alone…"
- Patients often ask clinicians what they think is best. Refrain from providing your own personal beliefs to avoid confusing or influencing patients and emphasise equipoise e.g.

  "I think that is a really difficult question because we don't genuinely know which is the best option. We just don't have enough information to say which treatment will be best for which patient, which is why we're doing this study. Both treatments have their pros and cons and have been assessed as being suitable for you. I would be happy to recommend the study to my close friends and family."

Mills N, *et al. BMJ Open* 2024; 14:e079108. doi: 10.1136/bmjopen-2023-079108

Exploring patient preferences

- It can be common for patients to arrive at a consultation with an expectation/preference about what treatment they would like. It is still important to discuss the study fully so that patients can make an informed decision about treatment options and study participation.
- **Strategies for responding to preferences:**
  - Acknowledge their preference and open up the conversation – *"Ok, but…"* is a good way to ensure this, e.g.
    "Ok it's great that you've read up on the surgery, but let's just consider what surgery means"
  - Remind patients to keep an 'open mind' until they've heard all information, e.g.
    "But what I'd like you to do is just keep an open mind whilst I run through the treatments. There may be aspects of treatment that you have not yet considered."
  - Explore the rationale behind their views and their understanding of the treatments. This can reveal misunderstandings or incorrect information, e.g.
    "I know that surgery is your preferred/least preferred option, what is it about surgery that draws you to it/concerns you?"
  - Balance their views, tailored to any concerns they may have, e.g.
    "Ok I accept what you say that surgery with chemotherapy is appealing because the cancer is removed, but we don't know if it makes any difference to your survival and the operation has associated risks. Chemotherapy alone is the current standard of care provided in both arms and does not have the associated risks of surgery."
  - Emphasise the position of uncertainty and not knowing which treatment option is best, e.g.
    "What you've got to remember is that both are good options that are suitable for you. If we knew which one was better we would recommend it."
  - Reassure patients about both treatments, e.g.
    "Neither of the treatments in the study are experimental, they have been used for years. The surgeon and oncologist have deemed them suitable for you."
- Continue with the conversation until you feel they are sufficiently open minded to consider either treatment option, in which case they are in an ideal position to be recruited. If they still have a clear preference, and you are satisfied that they are well-informed following the discussion, then they should not be recruited. This will minimise the risk of crossover.
- Patients preferences often dissipate following gentle exploration and balanced information.

Describing randomisation

- Randomisation is a familiar concept but it can be difficult to explain in a way that makes sense to patients in the context of a trial.
- Randomisation may not make sense to patients if they do not grasp *why* it is being done, so it is important to explain both the purpose and process of randomisation, e.g.
  "We don't know if it's better to have chemotherapy alone or with surgery so we want a fair comparison of the treatment options (purpose). We use a process of randomisation to produce two groups of patients that are similar except for the treatment received (process). This will enable us to do a fair comparison. You will have an equal chance of receiving chemotherapy alone or chemotherapy with surgery. If you or I chose then the groups are unlikely to be the same and the results may not be reliable."
- It is helpful to avoid using terms such as *'toss of a coin'* or *'decided by a computer'*. Metaphors have been viewed as being quite flippant for something so serious, and reference to a computer deciding has led to confusion that the computer is choosing the 'best' option for them.
- Randomisation can actually be a solution to uncertainty if the patient is unsure what to do.

Please ask any questions - thank you for your continued support!
